# Supplementary material for: Improved Production and Biophysical Analysis of Recombinant Silicatein-α
Source: Biomolecules. 2020 Aug 20;10(9):1209. doi: 10.3390/biom10091209 (PMC7565869; doi:10.3390/biom10091209)
Supplement: Supplementary file 1 [file biomolecules-10-01209-s001.pdf]

Supplementary Information

Improved Production and Biophysical Analysis of Recombinant Silicatein-α

Emily I. Sparkes [1,2], Rachel A. Kettles [1,2], Chisom S. Egedeuzu [1,2], Natalie L. Stephenson [1,2], Stephanie A. Caslin [1,2], S. Yasin Tabatabaei Dakhili [1,2], Lu Shin Wong [1,2,*]

[1] Manchester Institute of Biotechnology, University of Manchester, 131 Princess Street, Manchester M1 7DN, United Kingdom.

[2] Department of Chemistry, University of Manchester, Oxford Road, Manchester M13 9PL, United Kingdom.

* Correspondence: l.s.wong@manchester.ac.uk

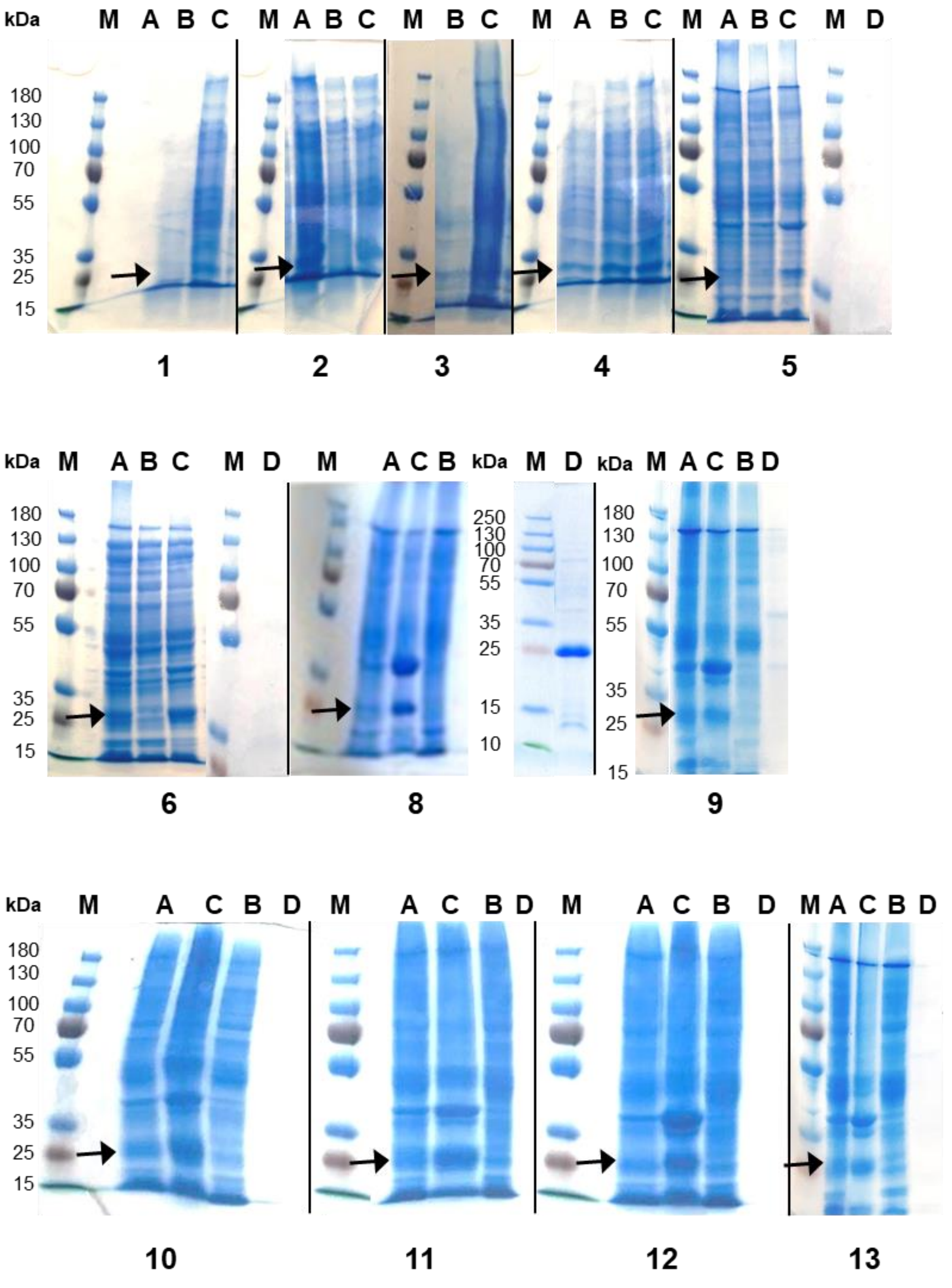

Figure S1. Images of SDS-PAGE gels demonstrating solubility analysis for His$_6$-Silα following lysis in various buffer mixtures (numbers below each image correspond to the buffers entries in Table 1). Lanes are labelled as follows: M = marker, A = total lysed protein, B = soluble lysis fraction, C = insoluble lysis fraction, D = post-IMAC fraction. Black arrows indicate presence of protein band of the desired size.

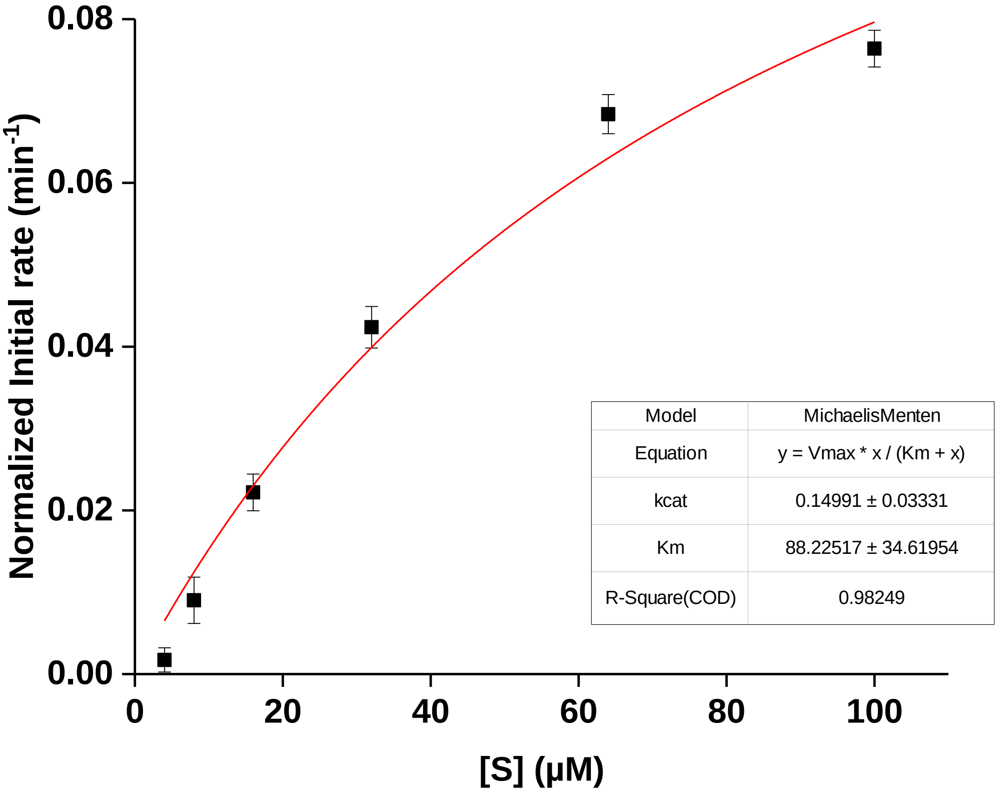

Figure S2. Michaelis-Menten plot of reaction velocity against TBDMS-ONp substrate concentration and best fit line. The error bars represent the standard deviations. The extracted parameters from the best-fit curve are shown in the inset, with standard deviations where appropriate.
